# Supplementary material for: Graphene memristive synapses for high precision neuromorphic computing
Source: Nat Commun. 2020 Oct 29;11:5474. doi: 10.1038/s41467-020-19203-z (PMC7596564; doi:10.1038/s41467-020-19203-z)
Supplement: Supplementary file 1 — Supplementary Information [file 41467_2020_19203_MOESM1_ESM.pdf]

Supplementary Information

Graphene Memristive Synapses for High Precision Neuromorphic Computing

Thomas F Schranghamer[1], Aaryan Oberoi[1], and Saptarshi Das[1,2,3,]*

[1]Department of Engineering Science and Mechanics, Pennsylvania State University, University Park, PA 16802, USA

[2]Department of Materials Science and Engineering, Pennsylvania State University, University Park, PA 16802, USA

[3]Materials Research Institute, Pennsylvania State University, University Park, PA 16802, USA

Supplementary Note 1:

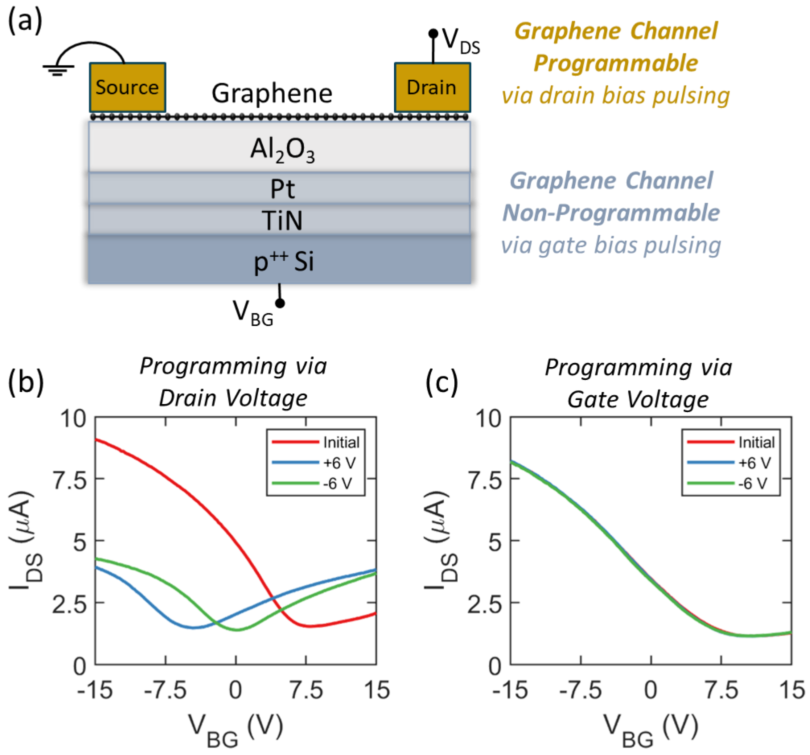

Figure 1: GFET schematic and programming terminal demonstration. *a.) Schematic of a GFET, consisting of a monolayer graphene channel and floating back-gate stack. Electrodes capable of supporting programming biases are identified. b.) V_{DS} programming results for positive and negative pulsing of magnitude 6 V and width 1 s. c.) V_{BG} programming results for the same pulsing scheme as in (b). Despite the large biases applied to the back-gate, no noticeable change in the transfer characteristics was seen, establishing that programming of GFETs is limited to bias pulses applied through the source/drain and thus is not a result of conductive filament formation/degradation like oxide-based memristors.*

Supplementary Note 2:

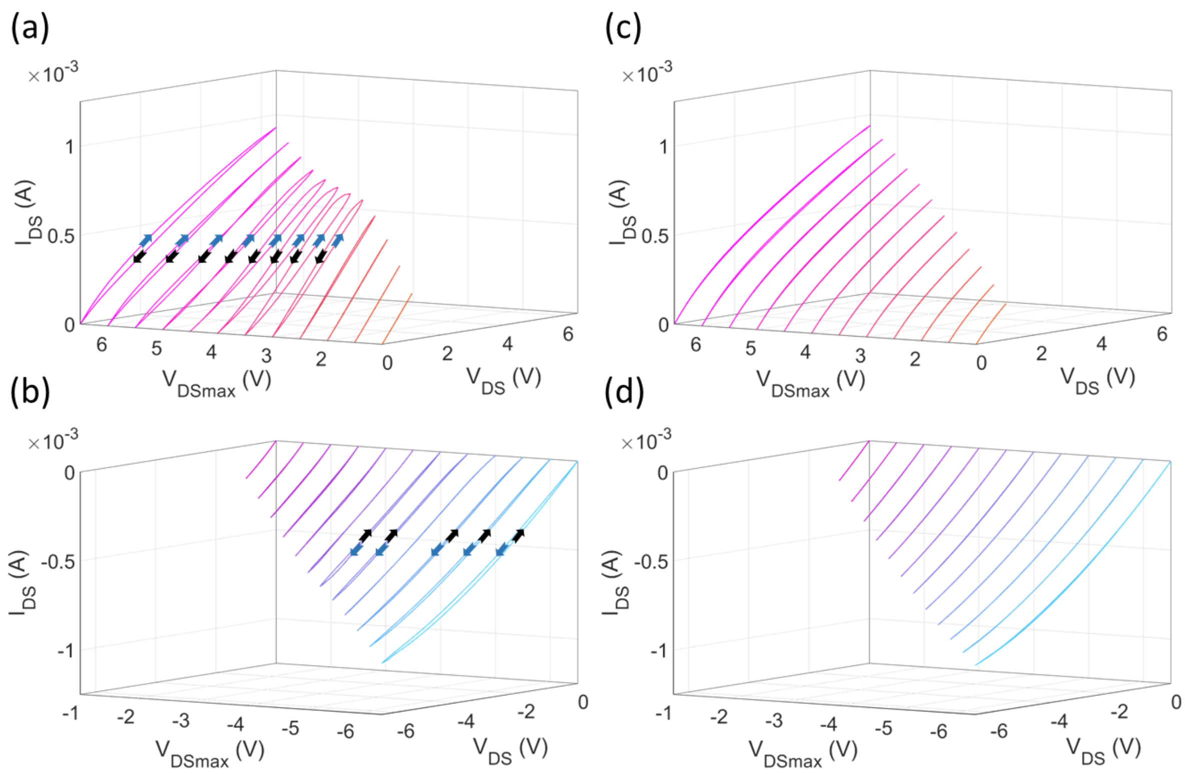

Figure 2: Hysteresis of PMMA-passivated graphene devices. *Output characteristics of as-fabricated GFETs following PMMA passivation at a back-gate bias of V_{BG} = 0 V for different V_{DS} sweep ranges (denoted by V_{DSmax}), from a) 1 V to 6.5 V and b) -1 V to -6.5 V in steps of 0.5 V. The arrows denote the sweep direction, with blue representing the forward sweep from 0 V and black representing the backward sweep from V_{DSmax}. Results are similar to those for unpassivated GFETs. As with the output characteristics of unpassivated GFETs shown in Fig. 1d and 1e, the hysteresis window initially increases with increasing V_{DSmax} and then reverses direction and starts to decrease. The presence of a passivation layer on the graphene channel establishes that the hysteresis switching, and thus the forming mechanism, does not rely on adsorbates (oxygen molecules, water molecules, etc.) on the graphene free surface. Note that this does not rule out interactions with adsorbates trapped at the graphene/Al_2O_3 interface. V_{DS} sweeping was repeated following that shown in (a) and (b), with the results being displayed in (c) and (d), respectively. Little-to-no hysteresis was seen, indicating a distinct state change in the GFETs and confirming the existence of the forming process. Due to the passivation layer, it can be assumed that the mechanisms are thus dominated by interactions at the graphene/Al_2O_3 interface.*

Supplementary Note 3:

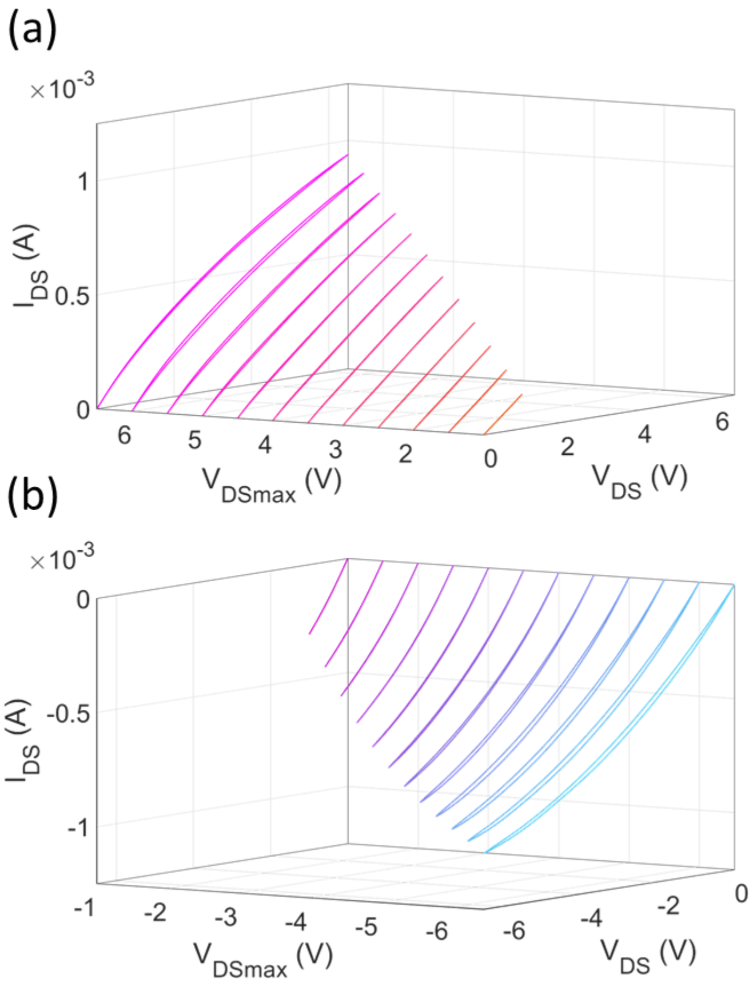

Figure 3: Hysteresis of reset graphene devices. *Output characteristics of GFETs at a back-gate bias of V_{BG} = 0 V for different V_{DS} sweep ranges (denoted by V_{DSmax}), from a) 1 V to 6.5 V and b) -1 V to -6.5 V in steps of 0.5 V. Measurements were taken following those shown in Fig. S2c and S2d, corresponding to (a) and (b), respectively, with the GFETs first being reset using a programming pulse of opposite polarity, magnitude 6.5 V, and duration 1 s.*

It is important to note the significant hysteresis at high V_{DSmax} demonstrated in Fig. 1d and 1e and Fig. S2a and S2b. Although the forming process was demonstrated to occur at lower V_{DSmax} values, as denoted by the switching of the sweep direction, the hysteresis remains high following forming. Based on the postulated forming mechanism discussed in the manuscript (i.e. the switching of water molecules at the graphene/Al_2O_3 interface from dissociative adsorption to molecular adsorption), the post-forming hysteresis should be negligible, akin to that seen in Fig. S2c and S2d.

To investigate the origins of the hysteresis at high V_{DSmax}, the GFETs used for the tests shown in Fig. S2 were subjected to large (6.5 V) programming pulses of negative and positive polarity immediately following the tests shown in Fig. S2c and S2d, respectively. The output characteristics of the GFETs were then measured for different V_{DS} sweep ranges, as in Fig. 1 and Fig. S2, with the results being shown in Fig. S3. The results show hysteresis greater than that displayed in Fig. S2c and S2d, though less than that displayed in Fig. 1 and Fig. S2a and S2b. This, in addition to the noticeable lack of sweep direction switching, indicates that the origin of the hysteresis is distinct from the forming mechanism seen in GFETs. Based on the memristive mechanism discussed in the manuscript (i.e. the reorientation of water dipoles at the graphene/Al_2O_3 interface), the hysteresis is believed to be a result of differences between the polarization of dipoles following each V_{DSmax} and the remanent polarization of the GFET (i.e. the polarization of the dipoles following the removal of the previous polarizing electric field). Due to initial disorder in the orientation of dipoles following the forming process[1], the hysteresis in the initial V_{DS} sweep testing, as shown in Fig. 1 and Fig. S2a and S2b, is relatively large. However, when the tests are redone without resetting the GFETs, as in Fig. S2c and S2d, the dipoles are already polarized, resulting in no hysteresis. When the devices are reset using programming pulses of opposite polarity (Fig. S3), the repolarization of the dipoles during following V_{DS} sweeps causes the hysteresis to return, albeit at a smaller magnitude than before the forming process, as would be expected of molecularly adsorbed water molecules when compared to dissociatively adsorbed molecules.

Supplementary Note 4:

As stated in the manuscript, the dominant interaction at the graphene/Al_2O_3 interface is believed to be that involving trapped water molecules. When the molecules are dissociatively adsorbed to the Al_2O_3 surface, the strong negative electric field generated has significant effect on the band structure of the graphene channel, effectively raising the valence band and inducing p-type doping. It has also been shown to induce interbanding between the graphene and O_S bands at the Dirac point. When the water molecules are shifted to a molecularly adsorbed state, the local electric field is removed, lowering the valence band and returning the Dirac point of the graphene to the Fermi level[1]. Experimentally, this is reflected by the transition of the GFETs to an ambipolar state from their initial p-type characteristics. While adsorbed water molecules at the interface are believed to be the dominant mechanism in the shown hysteresis and conductance switching, this does not rule out the existence of Al_2O_3 defects and trap states or other adsorbates, such as PMMA residue from the transfer and lithography processes, that may also affect the graphene channel. However, based on past studies of the effects of PMMA residue, such as heavy p-type doping, low conductivity due to carrier scattering, and large hysteresis from the introduction of trap states, PMMA residue should not be a major concern[2]. The rather moderate p-type doping demonstrated by the GFETs, as well as the fact that both it and the demonstrated hysteresis are removable by applying bias pulses through the drain, indicate that resist residues have very little effect, if any, on the graphene characteristics. While the argument may be made that these phenomena may be in part due to current-induced cleaning of the graphene channel[3], GFETs have been shown to return to their original p-type characteristics if left unused for long (on the order of weeks to months) periods of time and still remain programmable, ruling out PMMA residue as the dominant interaction. In addition, most trap states at interfaces with Al_2O_3 are attributed to dangling Al-O bonds at the

oxide surface[4]. As most of these states would be occupied by the adsorbed water molecules, the overall charge trapping should remain small.

<u>***Supplementary Note 5:***</u>

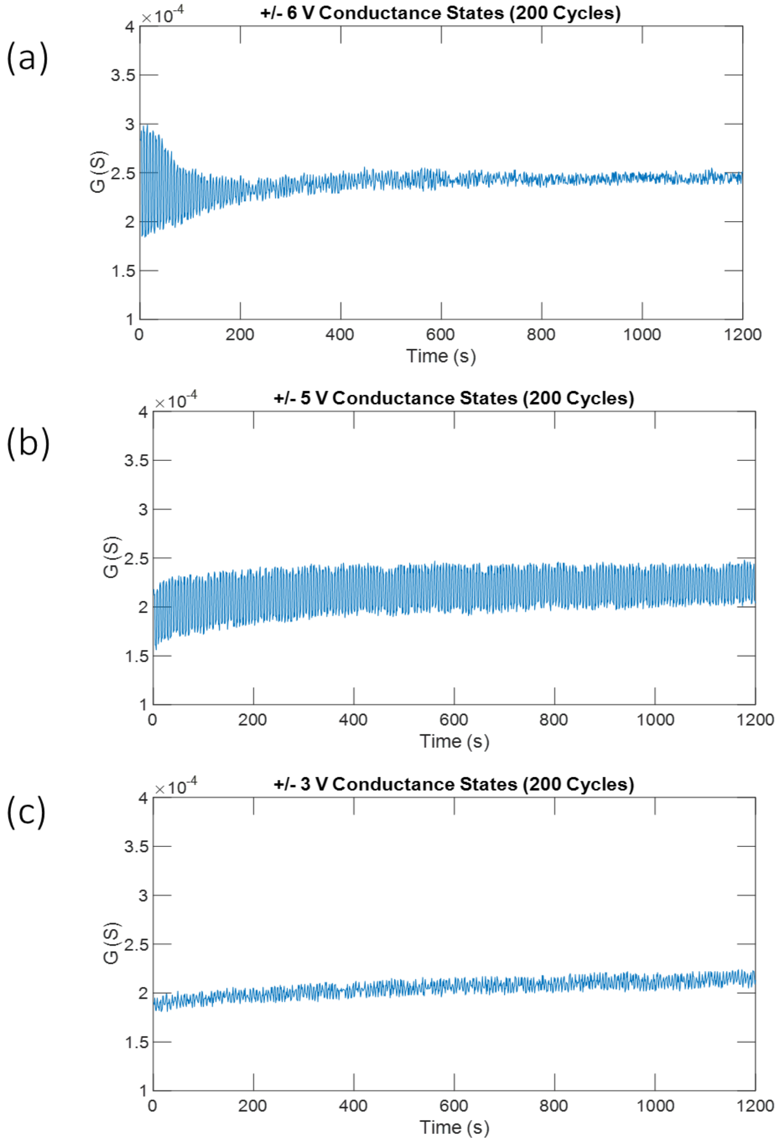

Figure 5: Conductance states over 200 cycles of SET and RESET pulses of different magnitudes. *a.) High magnitude V_{DS} = 6 V pulses display large initial memory ratios, but experience switching failure after only a few (< 100) cycles, indicating low endurance. b.) V_{DS} pulses of magnitude 5 V display both a relatively large difference in conductance and a switching endurance >200 cycles. c.) Low magnitude V_{DS} = 3 V pulses show reliable cycling but do not offer large memory ratio due to insufficient shift in the Dirac point and hence lack of difference in conductance states.*

Supplementary Note 6:

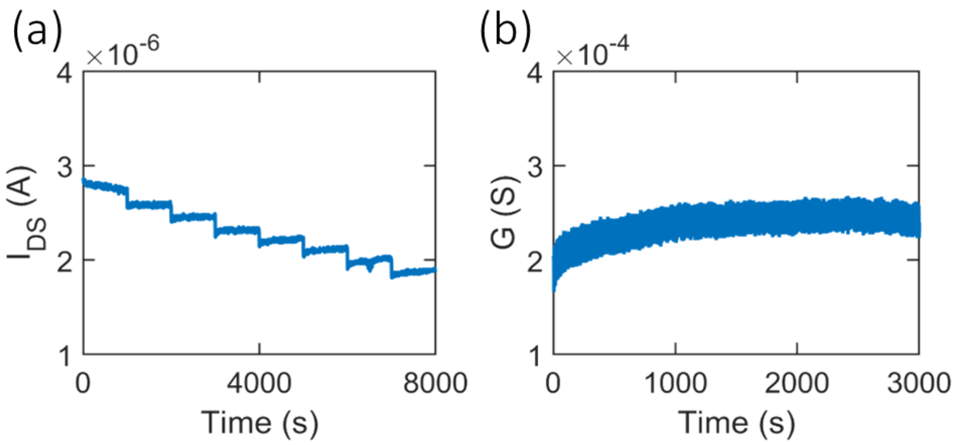

Figure 6: Long-term capabilities of GFETs. *a) Memory ratio and retention measured for a total duration of 1000 s for a GFET programmed into 8 memory levels using negative write pulses of step size 0.5 V and duration 1 s. Memory levels remain distinct for the entire read process, demonstrating good retention on par with a number of other published works concerning 2D material synaptic devices[5, 6, 7, 8]. b) Conductance states over 500 cycles of SET and RESET pulses of magnitude 5 V. The memory ratio remains consistent between the high and low conductance states over the entirety of the cycling process, signifying long term endurance. Endurance can be further improved by using lower pulse magnitudes during switching. As demonstrated in* ***Supplementary Note 8****, shorter channel length devices require smaller biases to induce conductance shifting, pointing towards higher endurance while still retaining reasonable memory ratios between the maximum and minimum conductance states.*

Supplementary Note 7:

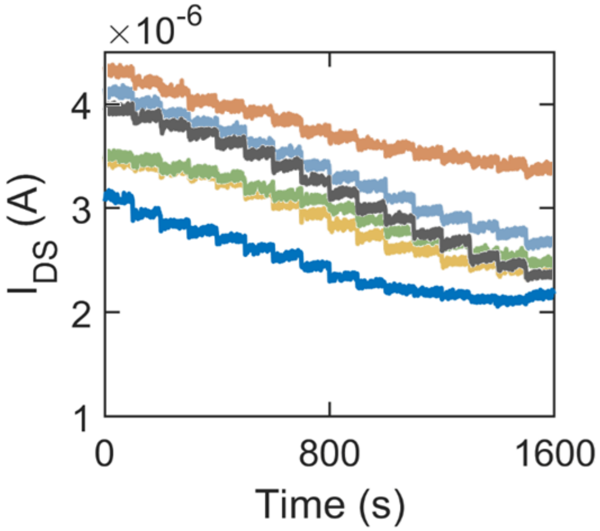

Figure 7: Effects of back-gate voltage on conductance states in GFETs. *The 16 conductance states (memory levels) obtained using write pulses (V_{DS}) with a step size of 0.125 V and duration 1 s are shown for different back-gate voltages: 0 V (blue), +5 V (black), +10 V (grey-blue), +15 V (orange), -5 V (yellow), and -10 V (green). At all non-zero back-gate voltages, the conductance for all 16 states is increased. Additionally, the memory ratio between neighboring states is controllably varied by changing the applied voltage, initially increasing for +/- 5 V and then decreasing at higher voltages as the device saturates. Notably, despite poor conductance switching (memory ratio) in the GFET at higher pulse magnitudes when V_{BG} = 0 V, performance is significantly improved for all back-gate voltages tested. It is apparent that applying different back-gate voltages to the GFETs during read and write operations allows for modulation, and even enhancement, of the memory levels achieved by GFETs.*

Supplementary Note 8:

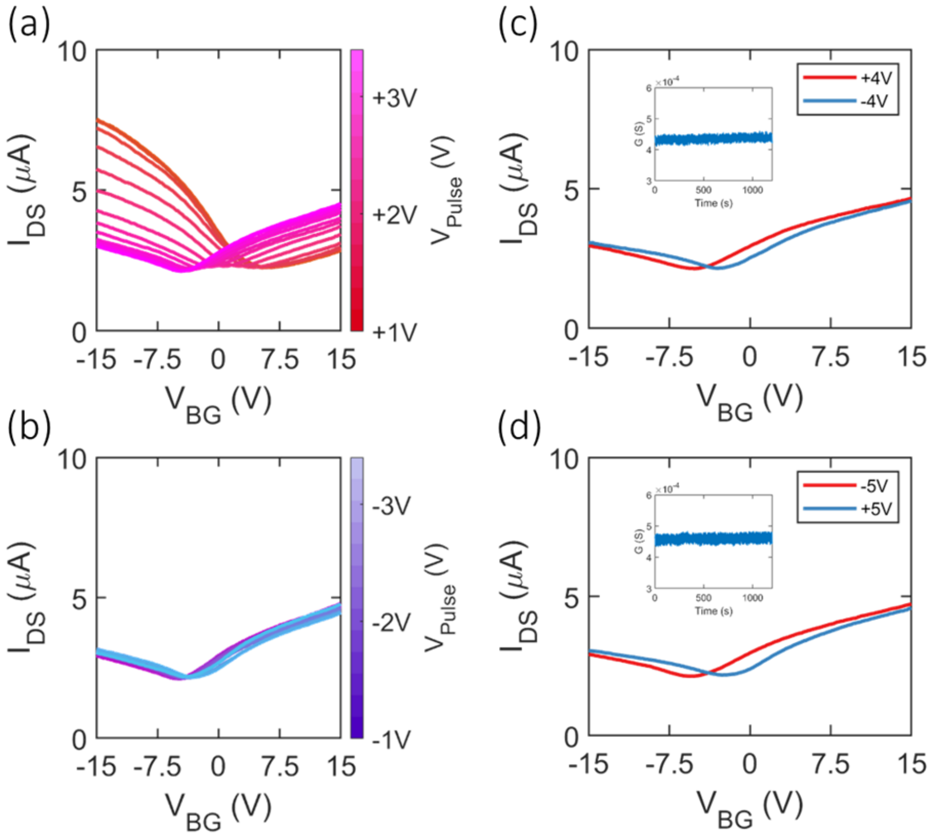

Figure 8: Forming, conductance switching, and endurance of scaled GFETs. *a) Transfer characteristics of a p-type GFET with channel length 400 nm and channel width 1 um when positive write voltage pulses of duration 1 s with increasing magnitude are applied, starting at +1 V and increasing to +3.4 V in steps of +0.2 V. The transition from p-type to n-type characteristics shown is similar to that demonstrated by the forming process displayed in Fig. 1, indicating the presence of memristive switching in scaled GFETs. The lower voltages needed to achieve forming for the scaled GFET indicates strong channel length/area dependence of the forming mechanism, implying that it is electric field dominated. b) Transfer characteristics of the same GFET when negative write pulses of the same magnitudes and step sizes as (a) are applied. The noticeable shift to more ambipolar characteristics from the n-type characteristics programmed in (a) confirms memristive switching in scaled GFETs. c-d) Transfer characteristics of the GFET for +/- c) 4 V and d) 5 V switching. Despite shifting beginning at lower voltages, the scaled GFET demonstrates the ability to be programmed up to the maximum voltages established for longer channel devices, indicating that shorter channel devices may be able to utilize a wider distribution of conductance states (memory levels). Insets display endurance testing over 200 cycles for respective pulse magnitudes with no observable degradation.* V_{Dirac} *shifting and endurance results of (c) and (d) are comparable to those of longer channel devices, indicating that GFETs are able to be scaled without adverse effects on device characteristics.*

Supplementary Note 9:

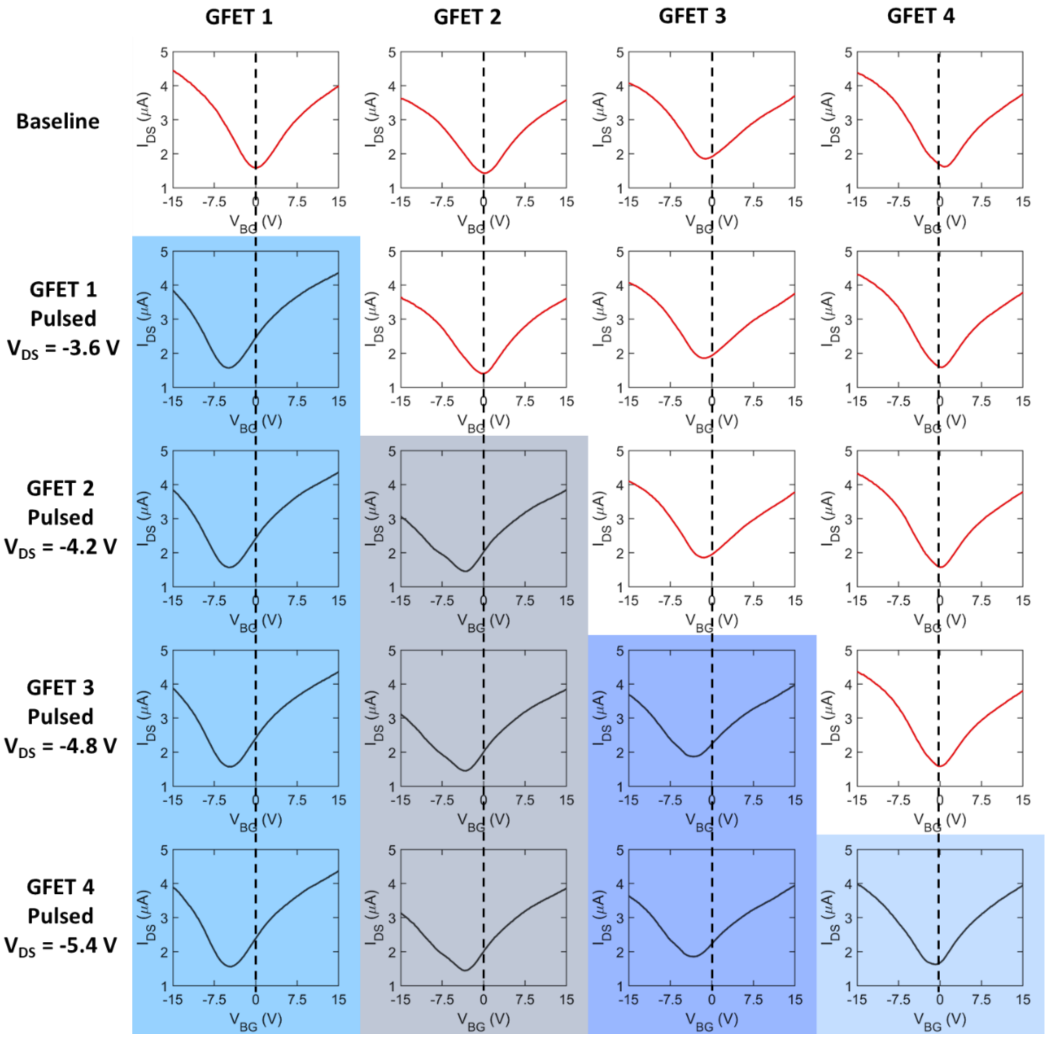

Figure 9: Demonstration of the programming isolation of conductance states in GFETs. *Due to the mechanisms behind traditional resistive memory (i.e. voltage pulsing through oxide and the formation of conductive filaments), sufficient electrostatic isolation is required for each memory device in an array to ensure that programming of one device does not affect the programmed conductance states of nearby devices. The* V_{DS} *pulsing demonstrated in this work, unlike gate voltage pulsing, operates directly through the channel of each GFET, therefore programming of GFET conductance states through* V_{DS} *pulsing should not affect nearby devices. To demonstrate this, a set of 4 GFETs (referred to as GFETs 1 – 4) arrayed in a row were independently subjected to programming pulses of varying magnitudes, with the transfer characteristics of all devices being analyzed following the programming of each device. All bias pulses utilized in this testing had a pulse time of 1 s. Following the initial setting of ambipolar characteristics in all GFETs, GFET 1 was programmed using* V_{DS} *= -3.6 V to a different conductance state. After programming of GFET 1, the transfer characteristics of the other 3 GFETs were measured to ensure that the programming of GFET 1 did not change the conductance states of the other devices. This process was then repeated with the application of negative voltage pulses of magnitudes 4.2 V for GFET 2, 4.8 V for GFET 3, and 5.4 V for GFET 4. A discrepancy that should be brought to attention is the slight negative and positive shifts in* V_{Dirac} *seen in the post-forming characteristics for GFET 3 and GFET 4, respectively. For the devices in question, it is possible that the slight positive/negative shifts of* V_{Dirac} *are due to the presence of non-water adsorbates, such as resist residue. For GFET 4, the slight shift of* V_{Dirac} *towards 0 V seen during the experiments where the device was not programmed suggests that the initial offset may have been due to incomplete population/depopulation of trap states at the graphene/*Al_2O_3 *interface, possibly from the water molecule adlayer, that was remedied by subsequent sweeping of the gate voltage.*

Supplementary Note 10:

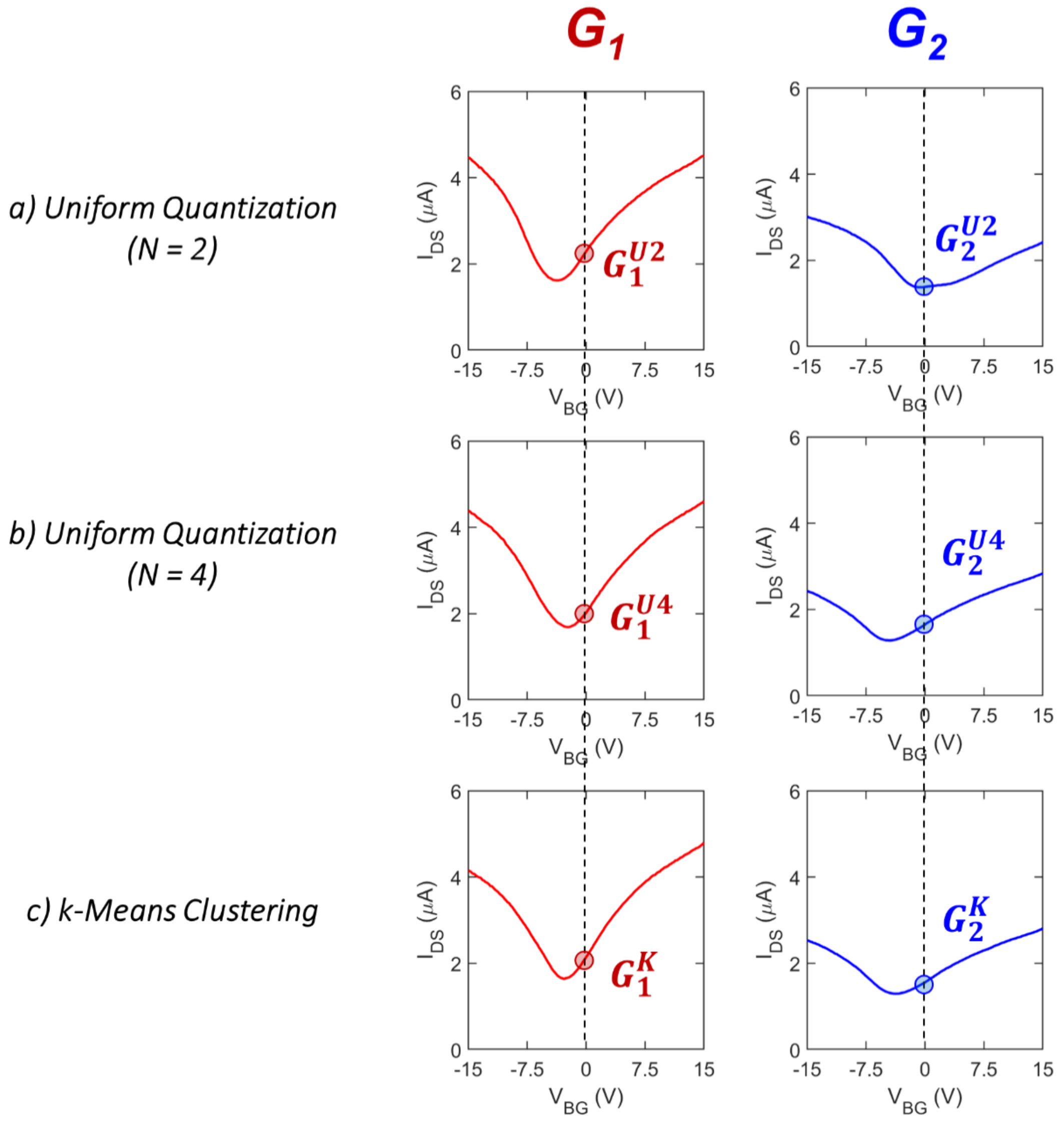

Figure 10: Transfer characteristics of GFET 1 and GFET 2 used for vector-matrix multiplication (VMM). *Architecture corresponding to different weight assignment schemes. a) Uniform quantization with N = 2 analog conductance states, b) uniform quantization with N = 4 analog conductance states, and c) k-means clustering based analog conductance states.*

Supplementary Note 11:

**Table 1: Memory characteristics of 2D materials-based synapses.** *A brief summary and comparison of the results of the proposed GFET memristive synapse to previous memristive synapses on 2D materials in a chronological order. More in-depth reviews can be found using Ref. 9 through 11.*							
**Authors**	**Device Structure**	**# of Memory States**	**Memory Ratio**	**Memory Retention**	**Memory Endurance (#) of Cycles)**	**Operating Speed**	**Switching Power/Energy**
Choi, Min Sup, et al. (2013)[5]	MoS_2/Graphene Heterostructure	2	~ 1E4	1400 s	> 110	100 µs	-
Choi, Min Sup, et al. (2013)[5]	Graphene/MoS_2 Heterostructure	2	~ 2	1200 s	> 100	1 ms	-
Tian, He, et al. (2017)[12]	2D $(PEA)_2PbBr_4$ Perovskite	4	1E2	1000 s	100	10 ms	400 fJ
Shi, Y., et al. (2017)[13]	Au/Ti/h-BN/Cu Electronic Synapse	2	1E2-1E4	-	-	20-200 µs	-
Sharbati, Mohammad Taghi, et al. (2018)[14]	Graphene/Li+ Electrochemical Synapse	250	7	13 hrs	> 500	10 ms	< 500 fJ
Sangwan, Vinod K., et al. (2018)[15]	Polycrystalline Monolayer MoS_2 FET	2	1E2	24 hrs	475	1 ms	-
Zhu, Jiadi, et al. (2018) (2018)[16]	Ion-gated WSe_2 FET	-	~ 1E5	5000 s	-	100 µs	30 fJ
Li, Da, et al. (2018)[17]	Multi-layer MoS_2 FET	2	-	-	6000	2-5 ms	-
Shi, Yuanyuan, et al. (2018)[18]	Multi-layer h-BN Synapse	2	25-1E4		> 1000	10 ns	6000 pW
Huh, Woong, et al. (2018)[6]	Vertically Integrated WO_{3-x} memristor and WSe_2/Graphene barristor	4	1E5	1000 s	1000	10 ms	0.1 nW
Zhu, Xiaojian, et al. (2019)[19]	Li_xMoS_2 Device	2	> 100	8000 s	1000	1 ms	-
He, Congli, et al. (2020)[7]	MoS_2 Multiterminal FET	6	1E5	> 1000 s	> 100	50 ns	7.3 fJ
**This work**	**Graphene FET**	**> 16**	**~ 12**	**> 1000 s**	**500**	**1 s**	**5 mJ**

Supplementary References

1. Cho SB, Lee S, Chung Y-C. Water Trapping at the Graphene/Al_2O_3 Interface. *Japanese Journal of Applied Physics* **52**, 06GD09 (2013).

2. Dan Y, Lu Y, Kybert NJ, Luo Z, Johnson AT. Intrinsic response of graphene vapor sensors. *Nano Letters* **9**, 1472-1475 (2009).

3. Moser J, Barreiro A, Bachtold A. Current-induced cleaning of graphene. *Applied Physics Letters* **91**, 163513 (2007).

4. Furchi MM, Polyushkin DK, Pospischil A, Mueller T. Mechanisms of photoconductivity in atomically thin MoS2. *Nano Letters* **14**, 6165-6170 (2014).

5. Choi MS, *et al.* Controlled charge trapping by molybdenum disulphide and graphene in ultrathin heterostructured memory devices. *Nature Communications* **4**, 1624 (2013).

6. Huh W, *et al.* Synaptic Barristor Based on Phase-Engineered 2D Heterostructures. *Advanced Materials* **30**, e1801447 (2018).

7. He C, *et al.* Artificial Synapse Based on van der Waals Heterostructures with Tunable Synaptic Functions for Neuromorphic Computing. *ACS Applied Material Interfaces* **12**, 11945-11954 (2020).

8. Vasicek Z, Mrazek V, Sekanina L. Automated Circuit Approximation Method Driven by Data Distribution. In: *2019 Design, Automation & Test in Europe Conference & Exhibition (DATE)*. IEEE (2019).

9. Seo S, *et al.* Recent Progress in Artificial Synapses Based on Two-Dimensional van der Waals Materials for Brain-Inspired Computing. *ACS Applied Electronic Materials* **2**, 371-388 (2020).

10. Wang CY, *et al.* 2D Layered Materials for Memristive and Neuromorphic Applications. *Advanced Electronic Materials* **6**, 1901107 (2019).

11. Wang S, Zhang DW, Zhou P. Two-dimensional materials for synaptic electronics and neuromorphic systems. *Science Bulletin* **64**, 1056-1066 (2019).

12. Tian H, *et al.* Extremely Low Operating Current Resistive Memory Based on Exfoliated 2D Perovskite Single Crystals for Neuromorphic Computing. *ACS Nano* **11**, 12247-12256 (2017).

13. Shi Y*, et al.* Coexistence of volatile and non-volatile resistive switching in 2D h-BN based electronic synapses. In: *2017 IEEE International Electron Devices Meeting (IEDM)*. IEEE (2017).

14. Sharbati MT, Du Y, Torres J, Ardolino ND, Yun M, Xiong F. Low-Power, Electrochemically Tunable Graphene Synapses for Neuromorphic Computing. *Advanced Materials*, e1802353 (2018).

15. Sangwan VK, *et al.* Multi-terminal memtransistors from polycrystalline monolayer molybdenum disulfide. *Nature* **554**, 500-504 (2018).

16. Zhu J, *et al.* Ion Gated Synaptic Transistors Based on 2D van der Waals Crystals with Tunable Diffusive Dynamics. *Advanced Materials* **30**, e1800195 (2018).

17. Li D*, et al.* MoS2 Memristors Exhibiting Variable Switching Characteristics toward Biorealistic Synaptic Emulation. *ACS Nano* **12**, 9240-9252 (2018).

18. Shi Y*, et al.* Electronic synapses made of layered two-dimensional materials. *Nature Electronics* **1**, 458-465 (2018).

19. Zhu X, Li D, Liang X, Lu WD. Ionic modulation and ionic coupling effects in MoS_2 devices for neuromorphic computing. *Nature Materials* **18**, 141-148 (2019).
